# In Vitro Wounding Models Using the Electric Cell-Substrate Impedance Sensing (ECIS)-Zθ Technology

**DOI:** 10.3390/bios8040090

**Published:** 2018-10-11

**Authors:** Andrea Y. Gu, Dan T. Kho, Rebecca H. Johnson, E. Scott Graham, Simon J. O’Carroll

**Affiliations:** 1 Department of Anatomy and Medical Imaging, School of Medical Sciences, Faculty of Medical and Health Sciences, University of Auckland, Auckland 1010, New Zealand; agu001@aucklanduni.ac.nz; 2 Centre for Brain Research, University of Auckland, Auckland 1010, New Zealand; d.kho@auckland.ac.nz (D.T.K.); rebecca.johnson@auckland.ac.nz (R.H.J.); s.graham@auckland.ac.nz (E.S.G.); 3 Department of Molecular Medicine and Pathology, School of Medical Sciences, Faculty of Medical and Health Sciences, University of Auckland, Auckland 1010, New Zealand

**Keywords:** ECIS, wound-healing assay, brain endothelium

## Abstract

Electric Cell-Substrate Impedance Sensing (ECIS) can produce reproducible wounding models by mechanically disrupting a cell monolayer. This study compared in vitro wound-healing using human cerebral microvascular endothelial cells (hCMVEC) with both single electrode (8W1E) and multiple electrodes (8W10E+) arrays. Measurements of hCMVEC migration and barrier functions were conducted, revealing variable levels of barrier disruption could be achieved by altering the duration and magnitude of the applied current. In all scenarios, the barrier (Rb) did not recover the strength observed prior to injury. Localization of junctional proteins following wounding were analyzed by immunocytochemistry. Following wounding, cell migration was generally faster on the 8W10E+ than the 8W1E array. Immunohistochemical analysis revealed non-viable cells remained on the 8W1E electrodes but not the 8W10E+ electrodes. However, viable cells partially remained on the 8W10E+ electrodes following wounding. In addition, the 8W10E+ electrodes demonstrated variation in cell loss across electrodes within the same well. This suggests the type of wounding is different on the two array types. However, our data show both arrays can be used to model incomplete barrier recovery and therefore both have potential for testing of drugs to improve endothelial barrier function. This is the first time that the possibility of using the 8W10E+ array as a wounding model is addressed. We highlight the differences in wounding produced between the two arrays, and can be used to study the underlying causes for impaired barrier function following CNS injuries.

## 1. Introduction

Compromised blood-brain barrier (BBB) function caused by neurological disease or injury leads to increased barrier permeability and infiltration of peripheral leukocytes into the CNS which can be detrimental [1,2]. Understanding these processes is crucial to developing interventions to protect or repair the BBB and, it is important to establish an in vitro human BBB model that reproduces barrier properties and thus advances our knowledge in the function of the human BBB in response to CNS injury. Currently, there is extensive research focusing on maintaining BBB function following CNS injuries using scratch-wound assays. However, scratch-wound assays are limited by poor consistency in the area of injuries [3]. The impedance-based Electric Cell-substrate Impedance Sensing (ECIS)-Zθ technology (Applied Biophysics) provides an alternative approach to produce in vitro wounding models.

The ECIS-Zθ is an in vitro system that monitors real-time cellular behaviours and movements via gold film electrodes and a number of different plate configurations are available [4]. These electrodes are 4.9 × 10^4^ µm^2^ and are connected to a larger counter electrode where a non-invasive alternating current flows with a frequency range of 10 to 10^5^ Hz [5]. The cell membranes essentially act as insulators. Consequently current flows unrestrained in the absence of cells and constrained once a cell monolayer is established. Changes in the current flow are measured as impedance (Z), which gives insight into two aspects of cellular behaviours and movements at different frequencies [6]. At low frequencies (<10,000 Hz), the cell bodies force the current to flow basolaterally or through the intercellular space between the cell borders. Therefore, resistance (R) is measured at low frequencies and provides information on the barrier integrity. Conversely, the opposition created by the cell membrane is relatively small at high frequencies (>10,000 Hz), so current flows capacitively through the cell bodies. Capacitance (C) is a measure of the electrode coverage by the cells and is indicative of cell migration, as well as cell monolayer disruption following injuries [5].

The ECIS set up can be used to wound a cell monolayer [7] using high current pulse produced via the electrodes. The severity of injury is dependent on the level of current and the duration of the application. The injured or dead cells then detach from the electrode surface which is measured as a rapid increase in the electrode capacitance and a reduction in the resistance. The system then returns to its normal operation and monitors the subsequent recovery as neighbouring cells migrate to fill the exposed electrode and re-establish a cell monolayer.

The ECIS arrays are available in a number of different electrode formats. The standard single electrode array, 8W1E, has been recommended and adapted in a number of in vitro wound-healing studies [7,8]. Wounding induced on the 8W1E array is reported to be confined to the surface of the ECIS electrode, therefore, provides a highly reproducible in vitro wounding model [7]. However, this implies that only a small proportion of the cell layer, approximately 0.1% cells in an area of 0.8 cm^2^, can be wounded and monitored. The multiple electrodes array, 8W10E+, consists of 40 electrodes which allow 40 times more cells to be wounded and monitored simultaneously. Measuring across multiple electrodes may give a more accurate idea of wound recovery. Because of the relatively high number of cells compared to the 8W1E, impedance fluctuations due to micromotion are smoothed out so as to not obscure subtle changes in impedance due to the experimental conditions.

This current study highlights the similarities and differences in injuries produced with the two types of arrays. Impaired recovery of the endothelial barrier strength was observed with both 8W1E and 8W10E+ arrays. A previous study has suggested that complete recovery of the impedance readings was prevented by dead cells that failed to detach from the electrode surface [7]. In this study, we found that in contrast to the 8W1E array, wounding on the 8W10E+ array induced complete detachment of the dead cells, and yet recovery was not complete. Unlike the 8W1E, a portion of the viable cells remained on the electrodes due to incomplete wounding. In addition, variation in the degree of injuries produced across multiple electrodes on the 8W10E+ array is addressed in this study.

## 2. Materials and Methods

### 2.1. Cell Culture

Given the complex structure of the BBB neurovascular unit, a monolayer model of microvascular endothelial cells is used as a simplified in vitro BBB model in this study. The human cerebral microvascular endothelial (hCMVEC) cell line was purchased from Applied Biological Materials (cat#T0259, ABM Good, Richmond, BC, Canada), and has been extensively characterized in terms of junctional protein expression and transendothelial electrical resistance (TEER) (O’Carroll et al., 2015; Wiltshire et al., 2016). The hCMVECs were maintained in M199 media (cat#11150-067, Gibco) supplemented with 10% FBS (cat#12203C-500ML, Sigma-Aldrich, St. Louis, MO, USA), 1 ng/mL hEGF (cat#PTAF10018B50, PeproTech, Rocky Hill, CT, USA), 3 ng/mL hFGF (cat#PTAF10015100, PeproTech), 10 μg/mL heparin (cat#H-3393, Sigma-Aldrich), 1 μg/mL hydrocortisone (cat#H0888, Sigma-Aldrich), 2 mM GlutaMAX (cat#305050-061, Thermo Fisher Scientific, Waltham, MA, USA) and 80 μM dibutryl cAMP (cat#D0627, Sigma-Aldrich). Cells were cultured under a 5% CO_2_ humidified condition at 37 °C and grown until reaching 80–90% confluency. Cells were used between passage 8 and 20.

### 2.2. Electric Cell-Substrate Impedance Sensing (ECIS) Wounding

ECIS wound-healing assays were conducted using the 8 well ECIS arrays (8W10E+, PC; 8W1E, PET) via the ECIS-Zθ station. The arrays were treated with 10 mM L-cysteine (cat#C7352-25G, Sigma-Aldrich) followed by coating with Collagen Type I (cat#A1048301, Thermo Fisher Scientific) at 1 μg/cm^2^. To sterilize and clean the gold electrodes the arrays the electrical stabilization command in the ECIS software was used. The hCMVECs were seeded onto the arrays at a density of 60,000 cells/cm^2^ in 500 μL of M199 growth media. ECIS was conducted using the multiple frequency/time (MFT) option to record the impedance measurements over a broad spectrum of frequencies. The hCMVECs were incubated for 48 h until impedance signals stabilized, indicating a confluent monolayer and a functional barrier had formed. The aim of this model is to be able to test interventions to strengthen an injured endothelial cell barrier. Wounding was carried out to obtain a “moderate” level of recovery of Rb that was similar between the two systems. To obtain a similar recovery profile between the two arrays, the wounding parameters were selected on the basis of the current level and its duration. For the 8W10E+ array, the maximum level of wounding current is 6500 uA in the manufacturer’s setting to avoid damage to the electrodes. However, for the 8W1E array, the level of wounding current cannot exceed 3000 uA in the manufacturer’s setting to avoid damage to the electrodes, hence only the duration of wounding was changed. Three levels of wounding parameters with different severities (Table 1) were tested. Following wounding, the impedance measurements were monitored for up to 96 h to monitor the recovery of the wounded hCMVECs.

### 2.3. Mathematical Modelling

A mathematical model can be applied to the impedance measurements in order to determine the endothelial barrier resistance (Rb) of the hCMVECs as function of the time and injuries. The mathematical model is applicable based on the assumptions of a confluent monolayer of circular cells with unchanged radius and densities, in addition, current must flow radially between the cells and the culture substrate [5]. Using the ECIS modelling software (Applied Biophysics), the multiple frequencies impedance measurements of a blank electrode, wells containing culture medium only, were calculated to exclude impedance changes due to the surface substrates. Data were analysed using GraphPad Prism 7 software, and were presented as the mean ± S.D.

### 2.4. Immunocytochemistry

Expression and localization of junctional proteins were analyzed by immunocytochemistry. The hCMVECs were fixed at 0 h, 2 h, 4.5 h, 24 h, and 48 h following wounding with 4% PFA and permeabilized with 0.1% Triton X-100 in PBS solution. Cells were incubated at room temperature with primary antibodies (refer to Table 2). Cells were then incubated with AlexaFluor-488 conjugated goat anti-mouse IgG (cat#A11029, Invitrogen, Carlsbad, CA, USA) at a dilution of 1:400 and AlexaFluor-488 conjugated goat anti-rabbit IgG (cat#A11034, Invitrogen) at a dilution of 1:1000, respectively. Cells were counterstained with Hoechst nuclear stain (cat#33342, Thermo Fisher Scientific) and ActinRed 555 ReadyProbes Reagent (R37112, Thermo Fisher Scientific).

### 2.5. Cell Viability Assay

At 10 min after ECIS wounding, the NucBlue^®^ Live and NucGreen^®^ Dead reagents from ReadyProbes^TM^ Cell Viability Imaging Kit (R37609, ThermoFisher) were added to the wells as per manufacturer’s instructions. NucBlue^®^ Live reagent stains all nuclei whilst NucGreen^®^ Dead reagent stains only nuclei of dead cells. Cells were then incubated for 15 min under culture conditions before microscopy analysis.

### 2.6. Cell Imaging

Images were acquired with a Zeiss Axioplan 2 Upright Fluorescence Microscope and a Zeiss Laser Scanning Microscope (LSM) 710 Inverted Confocal Microscope. Images were acquired at 5×, 20× and 40× magnification. Confocal images were collected as a series of 6–8 Z-stack images at 0.4–0.8 μm. Images were then processed using the MetaMorph Image Acquisition/Analysis 7.8.3 and Zeiss Zen Microscope software (blue edition), and were quantified using ImageJ software in terms of staining intensity and the number of nuclei [9].

### 2.7. Statistics

Statistical analyses were performed using GraphPad Prism 7 software. Data were analysed using one-way ANOVA followed by uncorrected Fisher’s Least Significant Difference (LSD) test. Graphical representations of p values were as follows: *, *p* < 0.05; **, *p* < 0.01; ***, *p* < 0.001; ****, *p* < 0.0001; ns, *p* > 0.05.

## 3. Results

### 3.1. Establishing a Wounding Model with the 8W10E+ Array

**Figure 1:**
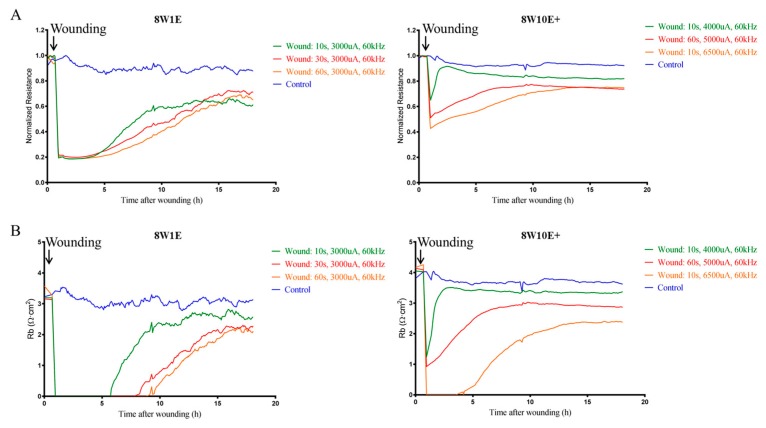
Comparative analysis of real-time endothelial barrier integrity following wounding on the 8W1E and 8W10E+ arrays. (**A**) Time-course of normalized resistance measurements following wounding on the 8W1E and 8W10E+ arrays; (**B**) Time-course of the modelled Rb, the measurement of the endothelial barrier resistance on the 8W1E and 8W10E+ arrays. The hCMVECs were seeded at 0 h at a density of 60,000 cells/cm^2^ on both 8W1E and 8W10E+ arrays. Wounding was applied at 48 h post-seeding. Three levels of wounding current are represented by green, red, and yellow lines, respectively. Blue line represents control cells that were not electrically wounded.

This study set out to establish whether it is feasible to develop a model of endothelial cell wounding using the ECIS 8W10E+ array, in order to test treatments to strengthen an injured barrier in future studies. The use of the 8W10E+ array for wound-healing assays has not been reported in the literature and therefore optimisation of the wounding parameters in comparison with the 8W1E was conducted. Wounding was applied at 48 h post-seeding when the hCMVECs formed functional barriers [10]. Figure 1A shows the injury and wound-healing measurements on both 8W1E and 8W10E+ arrays. Complete wounding was achieved on the 8W1E with a current of 3000 uA at 60 kHz for 10, 30, and 60 s. Resistance rapidly reduced following wounding, and progressively returned to 60–70% of the control levels. Recovery following 10 s of wounding was within 10 h, and was within 16 h following 30 or 60 s of wounding (Figure 1A). When the monolayer was re-established following injury, barrier resistance (Rb) was modelled to assess the integrity of the barriers. Incomplete recovery of Rb was observed following all levels of wounding; approximately 65% of that of the control cells (Figure 1B), this is in accordance with the results from a previous study [7].

We aimed to produce a level of the wounding that disrupted the endothelial barrier and required approximately 10–15 h for partial recovery on the 8W10E+ array. The recovery time varied between each of the injury conditions however, it is important to note that incomplete recovery was observed with all three levels of wounding (Figure 1A). A wounding current of 6500 uA at 60 kHz for 10 s allowed only 50% recovery of the Rb following injury (Figure 1B). It is possible that cells were unable to form functional barriers due to the severity of the injury. The Rb of cells that received a wounding current of 5000 uA at 60 kHz for 60 s, returned to approximately 65% of the levels of the control cells, which was similar to what we have observed on the 8W1E array (Figure 1B). Therefore, this level of wounding was selected for comparing the two arrays as this would be a good potential model for testing barrier strengthening compounds.

### 3.2. Change in the Electrode Capacitance Following Wounding is Smaller on the 8W10E+ Array

**Figure 2:**
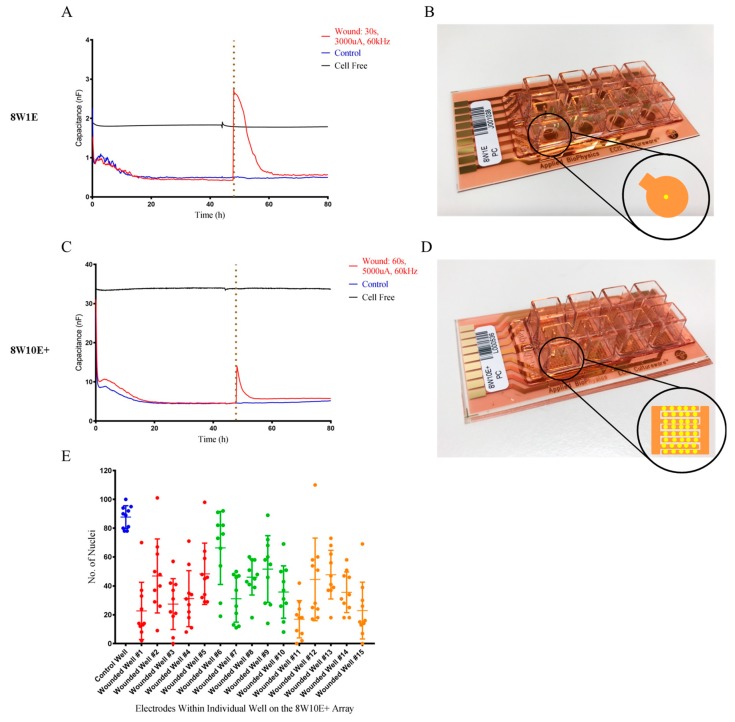
Changes in electrode capacitance following wounding on the 8W10E+ and 8W1E arrays. (**A**) Time-course of electrode capacitance measurements for the hCMVECs following wounding on the 8W1E array at 64,000 Hz; (**B**) 8W1E array (PC), single circular 250 μm diameter active electrode; (**C**) Time-course of electrode capacitance measurements for the hCMVECs following wounding on the 8W10E+ array at 64,000 Hz; (**D**) 8W10E+ array (PC), two sets of 20 circular 250 μm diameter active electrodes distributed along intergiditated fingers. The hCMVECs were seeded at 0 h at a density of 60,000 cells/cm^2^ on both 8W10E+ and 8W1E arrays. Red line represents a wounding current of 3000 uA at 60 kHz was delivered for 30 s to selected wells on the 8W1E array, and a wounding current of 5000 uA at 60 kHz was delivered for 60 s to selected wells on the 8W10E+ array. Blue line represents control cells that were not electrically wounded. Black line represents cell free electrode. The vertical line indicates the application of wounding current at 48 h; (**E**) Cell densities of individual electrodes on the 8W10E+ array at 0 h post-wounding. Data show 15 individual wells from three independent experiments. One-way ANOVA, uncorrected Fisher’s Least Significant Difference (LSD) test: *p* ≤ 0.0001, performed on 10 electrodes (every fourth electrode was selected from each well). Blue represents an unwounded well, where red, green, and orange each represents five individual wells from an independent experiment.

Following wounding, complete cell detachment on the 8W1E electrode occurred as indicated as the electrode capacitance was higher than that of the cell free electrode (Figure 2A). The small increase in the electrode capacitance on the 8W10E+ array indicated incomplete cell detachment (Figure 2C). Since there are 40 electrodes aligned along interdigitated fingers on the 8W10E+ array (Figure 2B), we asked whether the wounding pulse was evenly distributed across all 40 electrodes. Cell nuclei were fixed and stained immediately after wounding to assess cell coverage over the wounded electrodes in each well. A total of 10 electrodes from each well were analysed (where every fourth electrode was selected). Cell density on the wounded electrodes was significantly less than that on the control electrodes indicating cell detachment (Figure 2E). The majority of the wounded electrodes showed >50% cell detachment, and approximately 5% of the electrodes complete cell detachment whilst another 5% showed no cell detachment (Figure 2E). Moreover, variation in the degree of detachment was not related to the position of the electrodes. It is hypothesized that this variation is due to the delivery of wounding current. Supplementary Figure S1 shows the differences in cell detachment across 10 electrodes immediately following wounding. When cells on the first few electrodes have been wounded and detached, the opening of these electrodes became less resistant which allowed more current to flow through than those still covered by cells. The discrepancies in wounding current received between each electrode led to various degree of cell detachment, which was reflected in the small increase in the electrode capacitance of 8W10E+. Current flow was restricted to one electrode on the 8W1E (Figure 2B), therefore, complete wounding can be achieved.

### 3.3. Cellular Debris Remains Attached to the Wounded Electrodes on the 8W1E Array but Not the 8W10E+ Array

**Figure 3:**
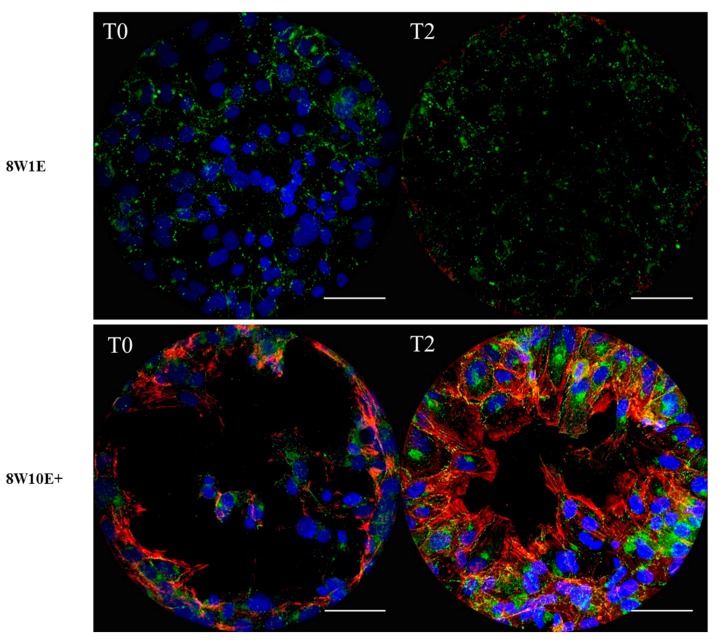
Differences in the wounding pattern between the 8W1E and 8W10E+ arrays. Representative images of the average degree of coverage from three independent experiments are shown. Images are a Z-stack composition between 0.4–0.8 μm at 0 h and 2 h post-wounding on different electrodes. The hCMVECs are labelled for Cx43 using rabbit polyclonal αCx43 antibody, visualized by goat α-rabbit Alexa Fluor 488 (green) at 40× magnification on the LSM 710 inverted confocal microscope. Actin filaments are stained with ActinRed 555 ReadyProbes Reagent (red). Nuclei are counterstained with Hoechst (blue). The hCMVECs were seeded at a density of 60,000 cells/cm^2^. A wounding current of 3000 uA at 60 kHz was delivered for 30 s to selected wells on the 8W1E array, and a wounding current of 5000 uA at 60 kHz was delivered for 60 s to selected wells on the 8W10E+ array. Scale bar = 50 μm.

To verify the degree of cell detachment following injury on each array, cells were labelled for actin and Connexin 43 (Cx43), a gap junction protein which is ubiquitously expressed in the endothelium [11]. Actin filaments were not observed on the electrode surface of the 8W1E array immediately following wounding (Figure 3). However, labelling of Cx43 protein and nuclei were observed on the 8W1E electrode at 0 h post-wounding (Figure 3). Therefore cell viability assays were performed to determine that the loss of actin was related to the cellular compromise. Supplementary Figure S2 shows that cells remaining on the electrode surface following wounding were non-viable. Additionally, 2 h post-wounding, cellular debris was still present which stained positively for Cx43 and there was a distinct lack of repopulation of the electrodes at 2 h post injury. (Figure 3). Although this observation contradictory to the corresponding electrode capacitance (Figure 2A), it implies that the debris remaining on the electrode surface did not constrain the current flow; therefore, the capacitance indicated a completely cell-free electrode. More importantly, the imaging reveals the present of cellular material, which may represent a physical barrier to the migratory healthy endothelial cells.

On the 8W10E+ array, however, there was no debris observed following wound-induced cell detachment (Figure 3) and individual electrodes had distinct regions devoid of any cellular debris or nuclei. Staining of Cx43 and the actin filaments were shown at the periphery of the wounding site (Figure 3). At 2 h post-wounding, cells can be seen repopulating the open electrode as the actin filaments were polymerized and protruded towards the centre of the wounding site (Figure 3). One thing to consider is that where live imaging is not available, each array has to be fixed for immunocytochemistry. Hence, images at various time points were acquired on different electrodes from different 8W10E+ arrays. However, Figure 2E shows that more than 50% cell detachment was observed on the majority of the wounded electrodes. Moreover, the rapid reduction in the electrode capacitance indicates cells were actively migrating, spreading and attaching to the electrode surface (Figure 2C).

### 3.4. Determining the Re-establishment of Barrier Integrity of the hCMVECs Following ECIS Wounding

**Figure 4:**
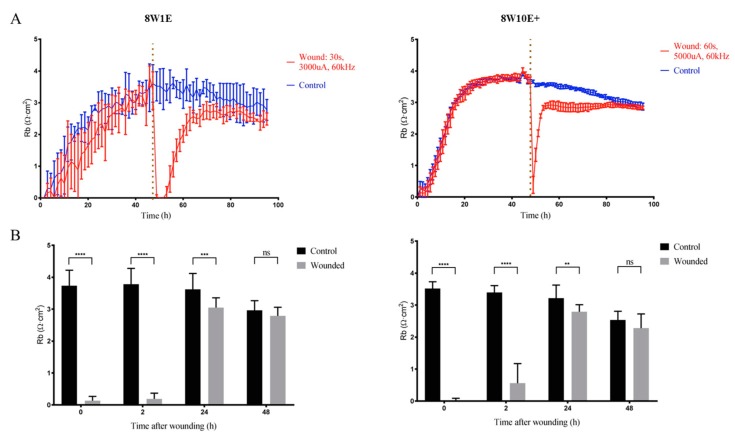
Changes in the barrier resistance over a time course of 48 h post-wounding on the 8W1E and 8W10E+ arrays. (**A**) Time-course of the modelled Rb, the measurement of the endothelial barrier resistance on the 8W1E and 8W10E+ arrays. The hCMVECs were seeded at 0 h at a density of 60,000 cells/cm^2^ on both 8W1E and 8W10E+ arrays. The red line represents a wounding current of 3000 uA at 60 kHz was delivered for 30 s to selected wells on the 8W1E array, and a wounding current of 5000 uA at 60 kHz was delivered for 60 s to selected wells on the 8W10E+ array. The vertical line indicates the application of wounding current at 48 h; (**B**) Statistical analysis of the modelled Rb post-wounding compared to those at unwounded state on the 8W1E and 8W10E+ arrays. Data show the mean ± S.D (n = 12 wells assessed from three individual experiments, One-way ANOVA, uncorrected Fisher’s LSD test: **p ≤ 0.01, ***p ≤ 0.001, ****p ≤ 0.0001, ns = not significant).

As aforementioned, Rb describes the barrier integrity of a monolayer [5], and thus Rb was assessed prior to wounding and after the monolayer was reformed. Both 8W1E and 8W10E+ arrays had similar Rb prior to wounding (≈4 Ω cm^2^) and post-wounding (≈3 Ω cm^2^). Neither array showed fully restored barrier strength over a time course of 48 h following the injury. In fact, barrier resistance was 10–20% less than that of the control cells until approximately 30 h post-wounding, where a reduction in the Rb of the control cell was observed (Figure 4A,B). As shown in Supplementary Figure S3, the modelled Rb of the control cells was reduced at a similar rate to those of the wounded cells after a second media change. This reduction in the modelled Rb at later stages is believed to be due to media evaporation over time. Notably, the signal fluctuation in the impedance measurements on the 8W1E array was diminished after wounding (Figure 4A). Fluctuation in the impedance signal is generally induced by cells moving on and off the electrodes [6]. This change was not seen on the 8W10E+ array, because the signal fluctuation was smoothed out as a result of averaged impedance measurements across 40 active electrodes (Figure 4A).

**Figure 5:**
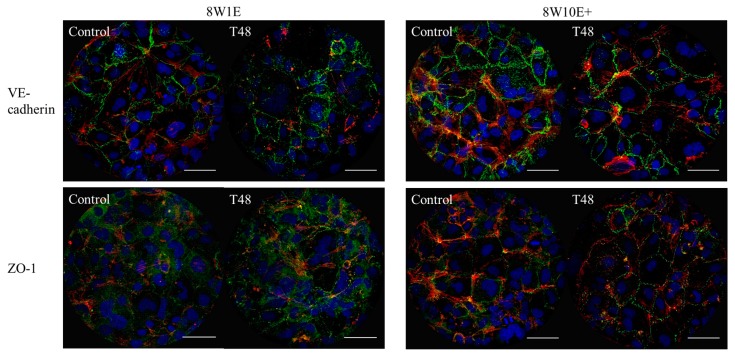
Changes in the hCMVEC morphology after wounding on the 8W10E+ and 8W1E arrays using the ECIS-Zθ system. Expression of the adherens junction protein, VE-cadherin, and the tight junction regulating protein, ZO-1, under control and wounded conditions in the hCMVECs on the 8W1E and 8W10E+ arrays. Representative images of three independent experiments are shown; images are a Z-stack composition between 0.4–0.8 μm at 48 h post-wounding and the control cells. The hCMVECs are labelled for VE-cadherin using mouse monoclonal CD144 antibody, ZO-1 using mouse monoclonal ZO-1 antibody, visualized by goat α-mouse Alexa Fluor 488 (green) at 40× magnification on the LSM 710 inverted confocal microscope. Actin filaments are stained with ActinRed 555 ReadyProbes Reagent (red). Nuclei are counterstained with Hoechst (blue). The hCMVECs were seeded at a density of 60,000 cells/cm^2^. A wounding current of 3000 uA at 60 kHz was delivered for 30 s to selected wells on the 8W1E array, and a wounding current of 5000 uA at 60 kHz was delivered for 60 s to selected wells on the 8W10E+ array. Scale bar = 50 μm.

**Figure 6:**
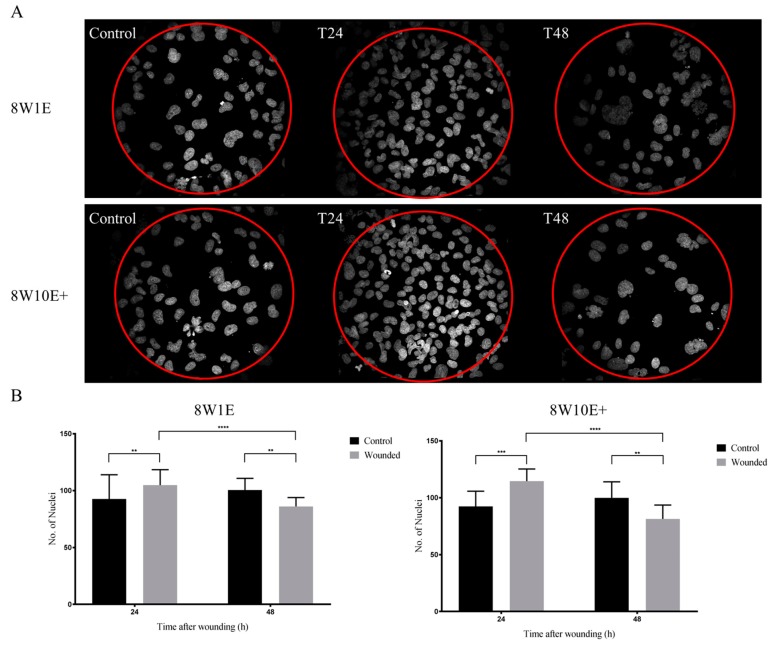
Cell densities following wounding on the 8W1E and 8W10E+ arrays. (**A**) Images shown are Z-stack composition between 0.4–0.8 μm at 24 h and 48 h post-wounding. Nuclei are stained with Hoechst (blue) and visualized at 40× magnification on the LSM 710 inverted confocal microscope. The red circle indicates area of electrode; (**B**) Nuclei count over wounded and unwounded electrodes at 24 h and 48 h post-wounding on the 8W1E and 8W10E+ arrays. Data show the mean ± S.D (*n* = 9 wells (10 electrodes was selected from each well) from three individual experiments, One-way ANOVA, uncorrected Fisher’s LSD test: **p ≤ 0.01, ***p ≤ 0.001, ****p ≤ 0.0001) The hCMVECs were seeded at a density of 60,000 cells/cm^2^. A wounding current of 3000 uA at 60 kHz was delivered for 30 s to selected wells on the 8W1E array, and a wounding current of 5000 uA at 60 kHz was delivered for 60 s to selected wells on the 8W10E+ array. Scale bar = 50 μm.

To assess if there were any differences in the recovery of the barrier following wounding between the two arrays, cells were immuno-labelled with the adherens junction protein, VE-cadherin (CD144), and the tight junction-associated protein, zonula-occluden-1 (ZO-1), which are both involved in regulation of, and are important determinants of, microvascular integrity in brain endothelial cells [12]. Re-establishment of the barrier integrity of the hCMVECs was analysed at 48 h post-wounding, a time when the hCMVECs have been shown to form functional barriers in normal plating conditions [10]. Defined junctional expression of VE-cadherin was observed at 48 h post-wounding on both 8W1E and 8W10E+ arrays (Figure 5). There appeared to be more cytoplasmic distribution of ZO-1 in the cells post-wounding when compared to the control cells, particularly on the 8W1E array (Figure 5). The cell density on the wounded electrodes at 48 h was significantly less than that of the unwounded electrodes (Figure 6). Interestingly, there was a large increase in cell density at 24 h post-wounding on both arrays, followed by a reduction between 24 and 48 h post-wounding (Figure 6). The confocal microscopy confirmed that the cells remained as an endothelial monolayer, as the same number of nuclei were present across all levels throughout (Figure 6A).

## 4. Discussion

We propose that the 8W10E+ array can be used for electrical wounding via the ECIS-Zθ system, and herein we highlight the advantages and disadvantages for its use. The 8W10E+ presents the advantage of having a greater coverage of the cell layer. In addition to the real-time impedance measurements, we have used fluorescent immunolabelling of junctional proteins to investigate the cell recovery following ECIS wounding. Our studies illustrate that there are similarities and differences in injury produced on the two ECIS arrays, the single electrode (8W1E) and the multiple electrodes (8W10E+) arrays.

Generally, the wound healing process involves four overlapping phases including haemostasis, inflammatory response, proliferation, and cytoskeletal remodelling [13,14]. The last phase is evident in both 8W1E and 8W10E+ wounding models. The impedance readings revealed that the electrical wounding damaged the endothelial cell layer. Drastic reductions in the barrier resistance of the hCMVECs were observed following wounding on both 8W1E and 8W10E+ arrays (Figure 1 and Figure 4). Wound-induced cell detachment was reflected by increases in electrode capacitance (Figure 2). Complete electroporation was induced on the 8W1E array, however, high-resolution confocal images revealed Cx43 protein debris and dead nuclei remained on the electrodes (Figure 3). Incomplete detachment of cellular structures from the 8W1E electrode surface has also been reported by Keese et al. (2004) and Gamal et al. (2015), where wounding was applied to normal rat kidney cells and human induced pluripotent stem cells of retinal pigment epithelium, respectively [7,15]. However, capacitance readings for these experiments were suggestive of a clean electrode. An explanation for this observation is that despite the remaining cellular structures, impedance is not detected as the unconstrained current flows through the damaged cell monolayer [7]. This is an important observation because cellular material left behind could potential impede or slow down the migration of cells moving into that space. The reformation of the barrier was generally slower on the 8W1E array, which is consistent with the cellular debris impeding or influencing reformation of the endothelial barrier. This presence of the debris is perhaps more indicative of wounding in vivo, where cellular debris would be expected.

Impedance readings indicating incomplete wounding was produced on the 8W10E+ array (Figure 1 and Figure 2). This was further confirmed in the degree of cell detachment seen across multiple electrodes within each well (Figure 2E and Supplementary Figure S1). However, there was no debris on the 8W10E+ electrodes following wounding-induced cell detachment. Viable cells, expressing junctional proteins along actin filaments, partially remained on the electrode surface (Figure 3). Taken together this suggests that the nature of injury occurring on the 8W10E+ is different to that occurring on the 8W1E. 

To further explore the differential wounding responses between the two ECIS arrays, the endothelial barrier resistance was assessed under both normal and wounded conditions. The endothelial barrier were re-established within 48 h following the injury, however, incomplete recovery of the barrier integrity was observed on both arrays in spite of the different types of injuries produced (Figure 4). On the 8W1E array, it has been reported that the debris remaining on the electrode surface prevented complete recovery of the barrier resistance [7]. The signal fluctuation, which is a measure of active cellular movements on and off the electrode surface [6], was reduced following wounding on the 8W1E array (Figure 4). Diminished fluctuation suggests that cells were not actively moving after injury, which is indicative of poor cell health. In addition, it is plausible that the increased cytoplasmic distribution of ZO-1 in the injured cells on the 8W1E was due to the debris. Consequently, cells surrounding the wounding site have to migrate over the remaining debris to re-establish a monolayer. On the 8W10E+ no cell debris is seen. Hence, cells adjacent to the site of injury would not be impeded from proceeding directly into cell migration. Nevertheless, impaired barrier integrity following wounding was also observed on the 8W10E+ array (Figure 3). This suggests that this is not entirely due to the presence of cell debris, as previously proposed [7]. These observations could be due to the nature of the cell line we have used. Abortive repair is commonly reported following CNS injuries as a result of excessive scarring and inability to repair damaged tissue, which subsequently leads to compromised BBB function [16]. Importantly, our data show both the 8W1E and 8W10E+ can be used to model incomplete barrier recovery and therefore both have potential for testing of drugs to improve the endothelial barrier function following injuries.

Both arrays showed a significant increase in cell density within 24 h of wounding (Figure 6). This increase was initially thought to be a result of wound closure. However, cell density was significantly reduced between 24 h and 48 h post-wounding on both ECIS arrays (Figure 6). Although increasing cell migration was triggered following wounding, the progressive reduction in cell density indicates that cells following wounding were viable for only a short period of time, which possibly suggests secondary injury or an inflammatory response. This observation is certainly worthy of further investigation with respect to in vivo pathological conditions.

We have demonstrated that wound-healing assays can be produced with the 8W10E+ array. The pathology of brain injuries causes highly heterogeneous tissue damage as a result of both primary and secondary injuries [17]. We suggest the variation in the degree of injuries produced with the 8W10E+ array could be used to model this heterogeneity that occurs in vivo. However, due to the increased number of electrodes in each well, the main concern for using the 8W10E+ array is the consistency of this variation. It is hypothesized that after the high current pulse was applied, some cells detach from the electrode surface faster than others. According to Ohm’s law (I = V/R), current travels through the path of least resistance [5]. Consequently, the exposed electrodes became less resistant, allowing more current to flow through. Less current was distributed to the cell-covered electrodes which led to partial or no cell detachment on a number of electrodes. Therefore, variation in wounding produced across the 40 electrodes on the 8W10E+ array must be taken into consideration when developing a wounding model.

## 5. Conclusions

In conclusion, this study addressed the possibility of using the multiple electrodes (8W10E+) ECIS array for wound-healing assays. Similar to the single electrode (8W1E) array, the 8W10E+ array is able to monitor cellular activities in response to injury in a real-time manner. Although this study has only employed a single cell line, these wounding models may be applied to other systems including primary cells. In addition, the two ECIS arrays have produced distinct types of injuries, which could be used for studies of different pathological conditions. The illustrated real-time wound-healing processes provide insight to the underlying causes of the impaired barrier function following CNS injuries, and allow future investigations for therapeutic interventions.

## Figures and Tables

**Table 1:** Details for levels of wounding applied to the 8W10E+ and 8W1E arrays using the ECIS-Zθ system.

	8W1E			8W10E+		
	Current (uA)	Frequency (kHz)	Time (s)	Current (uA)	Frequency (kHz)	Time (s)
Level of wounding	3000	60	10	4000	60	10
	3000	60	30	5000	60	60
	3000	60	60	6500	60	10

**Table 2:** List of primary antibodies used for immunocytochememistry.

Antibody	Host/Isotype	Class	Dilution	Incubation Time (h)	Company	Catalogue Number
Cx43	Rabbit/IgG	Polyclonal	1:400	2	Sigma-Aldrich	C6219
ZO-1	Mouse/IgG1	Monoclonal	1:100	1	Thermo Fisher Scientific	33-9100
VE-cadherin	Mouse/IgG1	Monoclonal	1:100	1	Santa Cruz	SC-9989

## Supplementary Materials

The following are available online at http://www.mdpi.com/2079-6374/8/4/90/s1.
