# Supplementary material for: In Vitro Wounding Models Using the Electric Cell-Substrate Impedance Sensing (ECIS)-Zθ Technology
Source: Biosensors (Basel). 2018 Oct 11;8(4):90. doi: 10.3390/bios8040090 (PMC6315451; doi:10.3390/bios8040090)
Supplement: Supplementary file 1 [file biosensors-08-00090-s001.pdf]

Supplementary Materials

In Vitro Wounding Models Using the Electric Cell-Substrate Impedance Sensing (ECIS)-Zθ Technology

Andrea Y. Gu [1,3], Dan T. Kho [2,3], Rebecca H. Johnson [2,3], E Scott Graham [2,3] and Simon J. O'Carroll [1,3,*]

1 Department of Anatomy and Medical Imaging, School of Medical Sciences, Faculty of Medical and Health Sciences, University of Auckland, Auckland 1010, New Zealand; a.gu@auckland.ac.nz
2 Department of Molecular Medicine and Pathology, School of Medical Sciences, Faculty of Medical and Health Sciences, University of Auckland, Auckland 1010, New Zealand; d.kho@auckland.ac.nz (D.T.K.); rebecca.johnson@auckland.ac.nz (R.H.J.); s.graham@auckland.ac.nz (E.S.G.)
3 Centre for Brain Research, University of Auckland, Auckland 1010, New Zealand
* Correspondence: s.ocarroll@auckland.ac.nz; Tel.: +64-9-923-9664

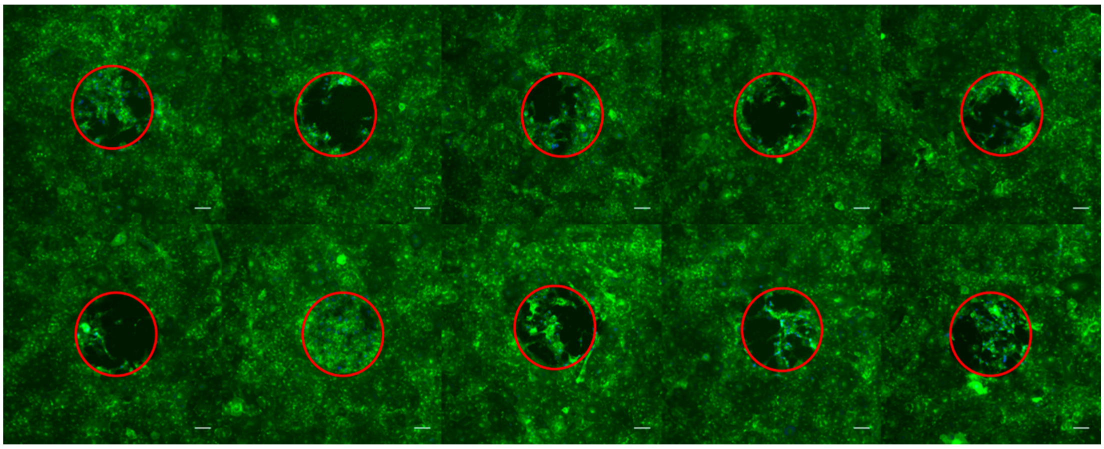

Supplementary Figure 1. Variation in cell detachment on the 8W10E+ electrode after wounding. The hCMVECs are labelled for Cx43 using rabbit polyclonal αCx43 antibody, visualized by goat α-rabbit Alexa Fluor 488 (green) at 40× magnification on the Zeiss Axioplan 2 Upright Fluorescence Microscope. Red circle indicates area of electrode. The hCMVECs were seeded at a density of 60,000 cells/cm^2. A wounding current of 5000 uA at 60 kHz was delivered for 60 s to selected wells on the 8W10E+ array. Scale bar = 50 µm.

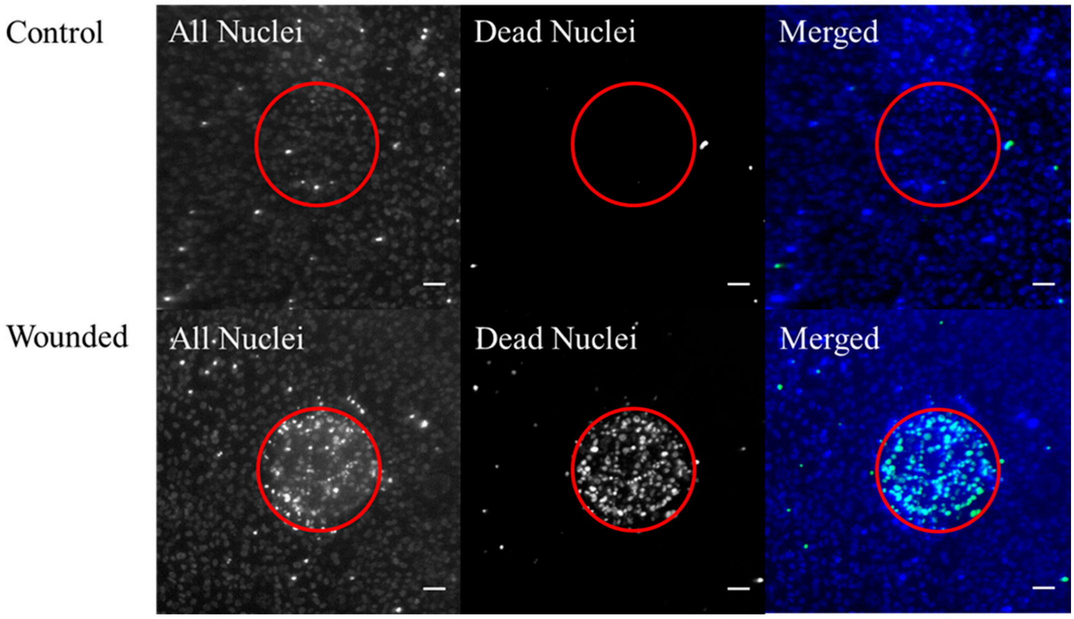

Supplementary Figure 2. Vital staining of the hCMVECs on the 8W1E electrode after wounding. Nuclei of the dead cells were stained with NucGreen® Dead reagent. Nuclei of all the cells were stained with NucBlue® Live reagent. The nuclei were imaged 30 min-post wounding at 5× magnification. Red circle indicates area of electrode. The hCMVECs were seeded at a density of 60,000 cells/cm². A wounding current of 3000 uA at 60 kHz was delivered for 30 s to selected wells on the 8W1E array. Scale bar = 50 μm.

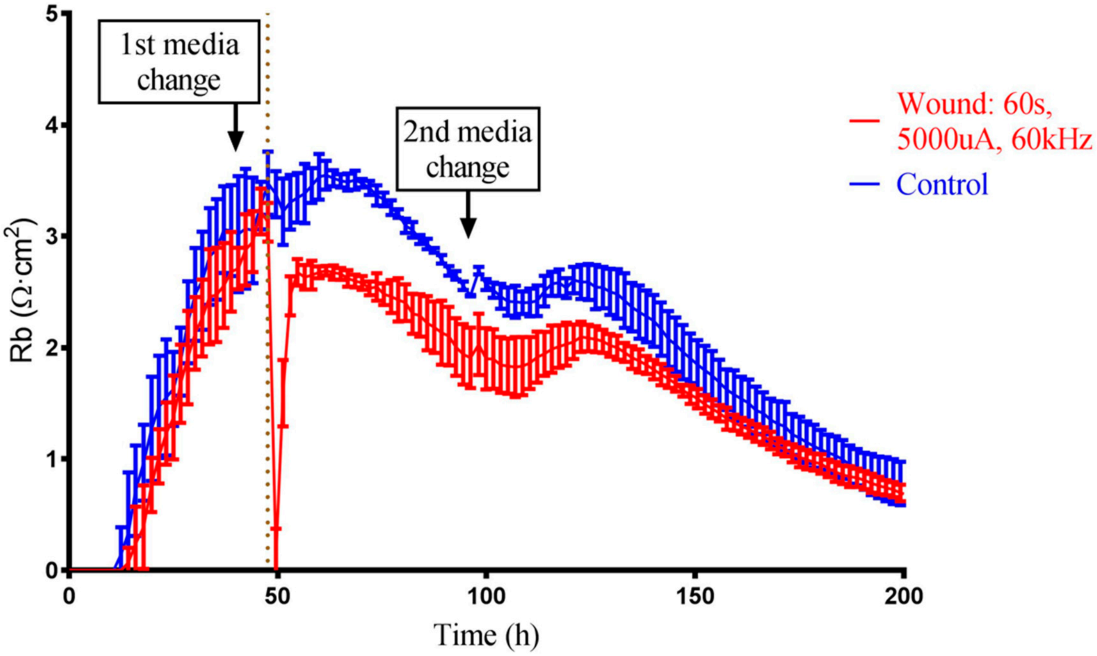

Supplementary Figure 3. Extended analysis of the barrier function post-wounding on 8W10E+ and 8W1E arrays using the ECIS-ZΘ system. Time-course of the modelled Rb, the measurement of resistance between cell-cell junctions in the endothelial monolayer on the 8W10E+ array. The hCMVECs were seeded at 0 h at a density of 60,000 cells/cm². Red line represents a wounding current of 5000 uA at 60 kHz was delivered for 60 s to selected wells. Blue line represents control cells that were not electrically wounded. Black line represents cell free electrode. First media change was at 44 h post-seeding. Second media change was at 96 h post-seeding. The vertical line indicates the application of wounding current at 48 h.
